# Transcriptome Analysis in Spleen Reveals Differential Regulation of Response to Newcastle Disease Virus in Two Chicken Lines

**DOI:** 10.1038/s41598-018-19754-8

**Published:** 2018-01-19

**Authors:** Jibin Zhang, Michael G. Kaiser, Melissa S. Deist, Rodrigo A. Gallardo, David A. Bunn, Terra R. Kelly, Jack C. M. Dekkers, Huaijun Zhou, Susan J. Lamont

**Affiliations:** 1 Department of Animal Science, Iowa State University, 806 Stange Rd, 2255 Kildee Hall, Ames, IA 50011 USA; 2 Population Health and Reproduction, School of Veterinary Medicine, University of California, Davis, CA 95616 USA; 3 Department of Animal Science, University of California, Davis, CA 95616 USA; 4 One Health Institute, School of Veterinary Medicine, University of California, Davis, CA 95616 USA

## Abstract

Enhancing genetic resistance of chickens to Newcastle Disease Virus (NDV) provides a promising way to improve poultry health, and to alleviate poverty and food insecurity in developing countries. In this study, two inbred chicken lines with different responses to NDV, Fayoumi and Leghorn, were challenged with LaSota NDV strain at 21 days of age. Through transcriptome analysis, gene expression in spleen at 2 and 6 days post-inoculation was compared between NDV-infected and control groups, as well as between chicken lines. At a false discovery rate <0.05, Fayoumi chickens, which are relatively more resistant to NDV, showed fewer differentially expressed genes (DEGs) than Leghorn chickens. Several interferon-stimulated genes were identified as important DEGs regulating immune response to NDV in chicken. Pathways predicted by IPA analysis, such as "EIF-signaling", "actin cytoskeleton organization nitric oxide production" and "coagulation system" may contribute to resistance to NDV in Fayoumi chickens. The identified DEGs and predicted pathways may contribute to differential responses to NDV between the two chicken lines and provide potential targets for breeding chickens that are more resistant to NDV.

## Introduction

Newcastle disease (ND) is a highly infectious disease that causes substantial economic losses to the global poultry industry. Although vaccination has greatly reduced outbreaks of the endemic disease, it cannot prevent the continuous evolution of ND virus (NDV)^1^ and some virulent strains can still survive in vaccinated chickens^2^. In addition, advanced vaccines and reliable infrastructure for transportation and storage of the vaccine are usually scarce in developing countries^3^, leading to mortality of chickens as high as 80% caused by the velogenic NDV that is endemic in these areas. To ameliorate the negative impact of NDV, alleviate poverty and improve food security in these countries, genetic selection and breeding approaches can be utilized, in addition to NDV vaccination, to enhance resistance of chickens to NDV.

The outcome of NDV infection depends on both the virulence of the NDV strain and the ability of chickens to resist to the virus. NDV strains can be classified from least to most virulent into lentogenic, mesogenic, and velogenic strains^4^. The severity of infection with the same NDV strain also varies between avian species and chicken breeds due to genetic variation^3^. There have been studies investigating host genetic regulation in response to different NDV strains^5,6^. However, few studies have reported gene expression responses to NDV in different chicken lines.

The ability of the host to expel pathogens and to limit their proliferation and shedding are components of resistance^7^. Two genetically distant inbred chicken lines – Fayoumi and Leghorn – provide a discovery platform to investigate genetic regulation of response and resistance to NDV. The Fayoumi line was derived from Egyptian fowl imported to the U.S. in 1954. Its robust resistance to some avian diseases, such as *Salmonella*^8^*, Eimeria*^9,10^, Marek’s disease^11^ and avian influenza^12^ has been reported. When the two chicken lines were infected with the same amount of a B1 type La Sota Lentogenic NDV strain, Fayoumis showed faster viral clearing than Leghorns from 2 to 6 days post infection (dpi) and produced more antibody in serum at 10 dpi compared to Leghorns^13^. Therefore, we hypothesize that Fayoumis are relatively resistant to NDV compared to Leghorns. Previously, we have compared the transcriptomic response to NDV in trachea between NDV-challenged and non-challenged chickens, as well as between the two chicken lines at 2, 6 and 10 dpi^13^. To gain a comprehensive understanding of regulation of immune response and resistance to NDV in chickens, however, we must also investigate gene expression response to NDV in other immune organs. The spleen is a major secondary immune organ where B and T cells mature in adult birds. It filters pathogens in the bloodstream, synthesizes antibody, and multiplies leukocytes^14^. The objectives of this study were to identify genes and pathways potentially regulating splenic response to NDV and to gain a more comprehensive understanding of genetic resistance to NDV through comparison of splenic gene expression between challenged and control groups, as well as between Fayoumi and Leghorn chickens.

## Results

### Differential expression identified by RNA-seq and validated by Biomark IFC

**Figure 1:**
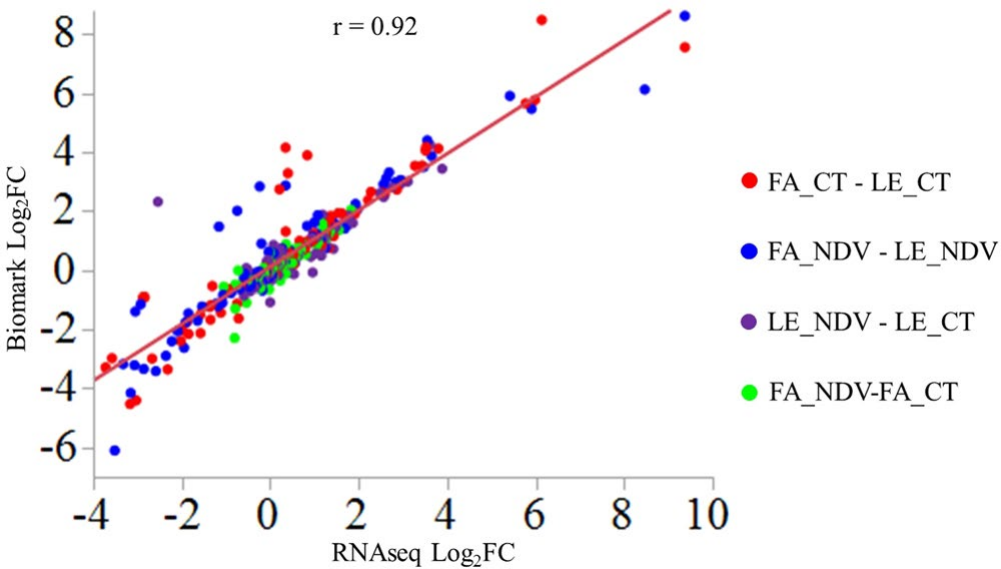
Validation analysis by Fluidigm Biomark assay of log_2_ fold change (Log_2_FC) of selected genes that were significant for different contrasts in RNA-seq analysis. Contrasts between different line by treatment combinations are marked in different colors and each combination is labeled as Line_Treatment (FA: Fayoumi, LE: Leghorn, CT: Control, NDV: Newcastle disease virus). Pearson correlation coefficient is labeled as “r”. Log_2_FC in Biomark assay equals −ΔΔCt for each comparison. Average cycle threshold (Ct) value for each group is the mean of samples in that group. Average expression of three housekeeping genes including *GAPDH*, *ACTB* and *HPRT1* were used for normalization of Ct values.

Four chickens in each line at each time were selected for RNA-seq study. Measurement of viral copy number in lachrymal fluid showed no virus in control groups but significant differences of NDV load among groups in NDV-challenged chickens (Table 1). The two chicken lines showed no significant difference in the viral copy number at 2 dpi, but Fayoumis showed larger decrease after 4 days, making it significantly lower than that in Leghorns. Millions of 100-bp single-end reads were obtained for each sample from sequencing of libraries constructed with RNA samples from spleen. Although there was large variation in the number of raw reads (from 8,957,210 to 18,183,922) among samples after filtering, similar percentages of reads (around 90%) of each sample were mapped to the *Gallus gallus* Galgal 5.0 reference genome in the Ensembl database. On average, expression of 17,020 genes were detected in each individual, accounting for 68% of the 24,881 annotated genes in the reference genome (Supplementary Table S1). Log_2_ fold change (log_2_FC) of 40 selected genes in different comparisons showed very high consistency between expression data obtained by Fluidigm Biomark qPCR and RNA-seq (correlation coefficient: r = 0.92), validating the differential expression identified by RNA-seq (Fig. 1).

**Table 1:** Viral copy number in the lachrymal fluid of NDV-challenged chickens in RNA-seq study.

Line	dpi	ID	Log_10_ (Viral Copy Number)	Mean ± SEM
Fayoumi	2	1561	7.10	7.08 ± 0.05^a^
Fayoumi	2	1581	6.95	
Fayoumi	2	1584	7.21	
Fayoumi	2	1593	7.06	
Leghorn	2	1509	6.81	6.62 ± 0.12^ab^
Leghorn	2	1520	6.60	
Leghorn	2	1537	6.79	
Leghorn	2	1540	6.29	
Fayoumi	6	1559	4.58	5.12 ± 0.38^c^
Fayoumi	6	1560	5.68	
Fayoumi	6	1569	5.86	
Fayoumi	6	1586	4.36	
Leghorn	6	1503	6.26	6.12 ± 0.07^b^
Leghorn	6	1535	6.05	
Leghorn	6	1546	6.22	
Leghorn	6	1549	5.94	

Note: Different letters (a–c) in the superscript means significant difference among groups. dpi: days post infection. SEM: Standard error of mean.

**Figure 2:**
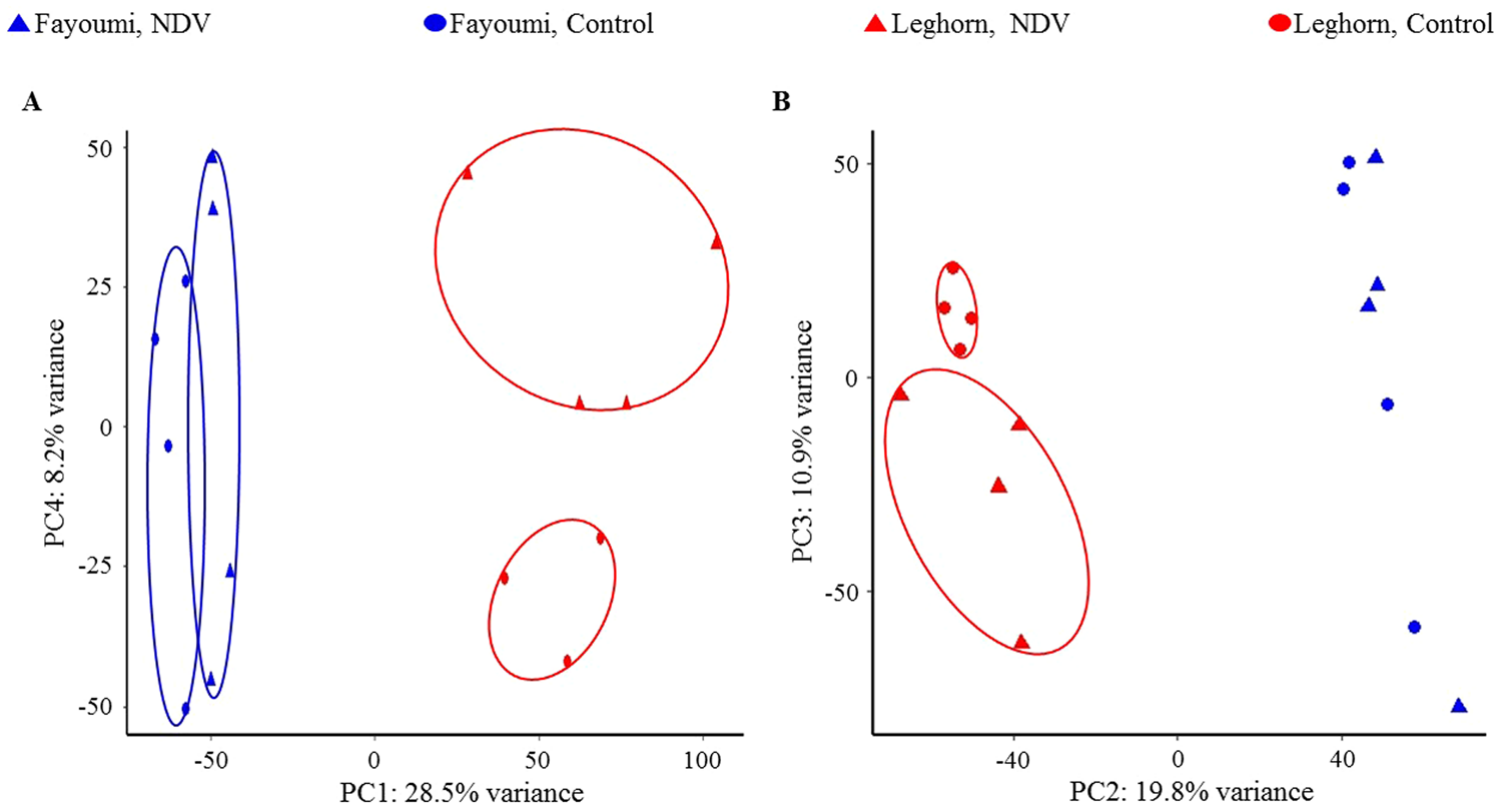
Principal component analysis (PCA) plots generated with ggbiplot in R showing variation and clustering of samples in different groups. (**A**) PCA plot for samples at 2 dpi. (**B**) PCA plot for samples at 6 dpi. Horizontal and vertical axis show two principal components that respectively explain variation between different lines and that between different treatments. Sample clusters in different groups are circled with ellipses. Different groups are represented in different shapes and colors: Fayoumi (blue), Leghorn (red), NDV (triangle), Control (circle).

The principle component analysis (PCA) plot showed clear separation of samples at 2 dpi between the genetic lines along principle component 1 (PC1) explaining over 28% of variance. The samples of different lines at 6 dpi also clustered well along PC2 explaining over 19% of variance. Clustering of samples at 2 dpi based on treatment was also explicit for Fayoumis along PC1 and Leghorns along PC4 (Fig. 2A). Clustering of samples at 6 dpi based on treatment was clear for Leghorns along PC3 which explained about 10% of variation, but treatment groups in Fayoumi samples were not separated by any principle component (Fig. 2B).

**Figure 3:**
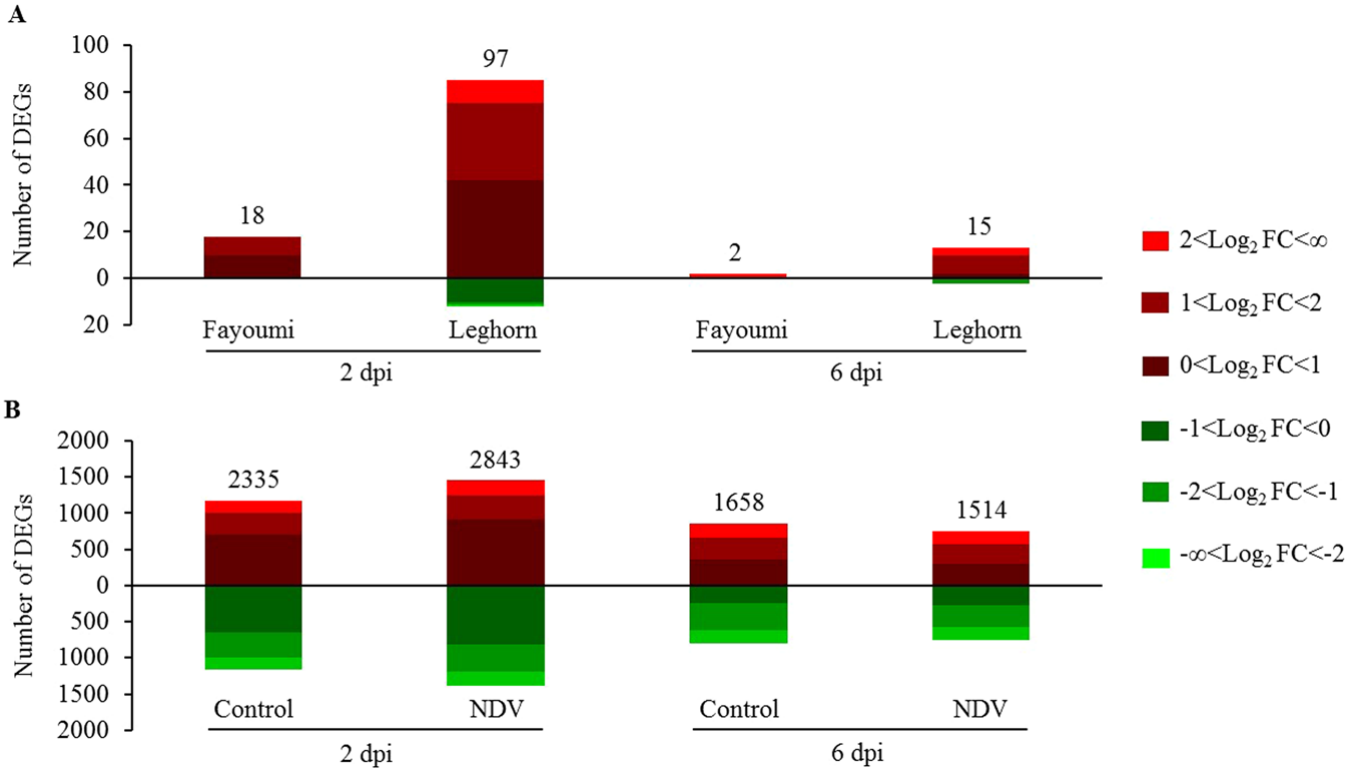
Number of significant differentially expressed genes (DEGs) at a false discovery rate <0.05 at 2 and 6 dpi. (**A**) Number of DEGs for NDV vs. Control comparisons within each line; (**B**) Number of DEGs for Fayoumi vs. Leghorn comparisons within each treatment. Upregulated and downregulated DEGs are represented in red and green color, respectively. DEGs within different range of Log_2_FC are shown in the color of different intensities.

The contrast between NDV-infected and non-infected groups showed that NDV challenge induced more upregulated DEGs than downregulated DEGs with a false discovery rate (FDR) less than 0.05 and the number of DEGs showed a large decrease from 2 to 6 dpi in both lines (Fig. 3A). However, even with the similar level of viral load in lachrymal fluid at 2 dpi, the number of DEGs was much higher in Leghorns than in Fayoumis at both time points (Fig. 3A, Supplementary Table S2). Fifteen annotated DEGs showed significant upregulation (FDR <0.05) in both lines at 2 dpi, and their fold changes were always slightly higher in Leghorns than in Fayoumis at both 2 and 6 dpi (Table 2). However, as immune system recover from viral challenge, only three of the 15 DEGs, including interferon induced protein with tetratricopeptide repeats 5 (*IFIT5*), myxovirus-resistance protein 1 (*Mx*) and radical s-adenosyl methionine domain containing 2 (*RSAD2*), were still significantly upregulated at 6 dpi in challenged Leghorns at 6 dpi, and none of these genes were upregulated in challenged Fayoumi at 2 dpi (Table 2). In our previous study investigating gene expression change in tracheal epithelium^13^, because we used Galgal4 reference genome rather than Galgal5, expression of 5 of the 15 DEGs were not detected. But the other 10 DEGs also showed significant up-regulation in challenged birds of both chicken lines at 2 dpi compared to non-challenged group (Table 2), indicating the ubiquitously important roles of these genes in regulating early immune response to NDV. Interestingly, all of these genes showed much higher fold change in trachea than in spleen in the NDV vs. control contrast in the same line at the same time point (Table 2).

**Table 2:** Genes showing common upregulation in response to NDV in both Fayoumi and Leghorn chickens.

Ensembl Gene ID	Gene Name	Log_2_FC (NDV vs. Control) (Spleen/Trachea)			
		Leghorn 2 dpi	Fayoumi 2 dpi	Leghorn 6 dpi	Fayoumi 6 dpi
ENSGALG00000016400	*RSAD2*	2.35*/6.62*	1.26*/6.19*	1.19*/2.45*	0.50/1.86
ENSGALG00000045085	*IFIT5*	3.11*	1.21*	1.28*	0.57
ENSGALG00000016142	*Mx*	3.61*/4.31*	1.18*/4.00*	1.87*/1.98*	0.59/1.72
ENSGALG00000038140	*DDX60*	2.43*	1.08*	1.26	1.08
ENSGALG00000028982	*CMPK2*	2.75*/3.77*	1.32*/3.70*	1.06/1.10	0.5/1.11
ENSGALG00000009479	*SAMD9L*	2.48*/7.63*	1.21*/6.65*	1.17/3.95*	0.32/3.9*
ENSGALG00000013723	*OASL*	2.96*	1.07*	1.16	0.44
ENSGALG00000013057	*USP18*	1.78*/4.55*	0.98*/4.37*	0.66/1.54*	0.35/1.69*
ENSGALG00000004859	*ZNFX1*	1.48*/2.26*	0.76*/1.92*	0.61/0.62	0.29/0.60
ENSGALG00000031916	*ZP1*	1.14*	0.65*	0.20	0.05
ENSGALG00000001558	*MOV10*	1.44*/3.68*	0.62*/3.30*	0.28/1.46*	0.20/1.23
ENSGALG00000003144	*TRIM25*	1.19*/2.25*	0.60*/2.01*	0.24/0.81	0.19/0.67
ENSGALG00000014603	*C1S*	0.82*/2.03*	0.61*/1.47*	0.56/0.74	0.23/0.62
ENSGALG00000045511	*PARP9*	0.88*	0.58*	0.10	−0.04
ENSGALG00000010560	*EIF2AK2*	0.92*/2.59*	0.51*/2.46*	0.40/0.88	0.20/0.57

Note: Log_2_FC means Log_2_ fold change. *Indicates that differential expression is significant (false discovery rate <0.05). Genes with observed data in trachea in our previous study^13^ have their log_2_FC shown as spleen/trachea.

**Figure 4:**
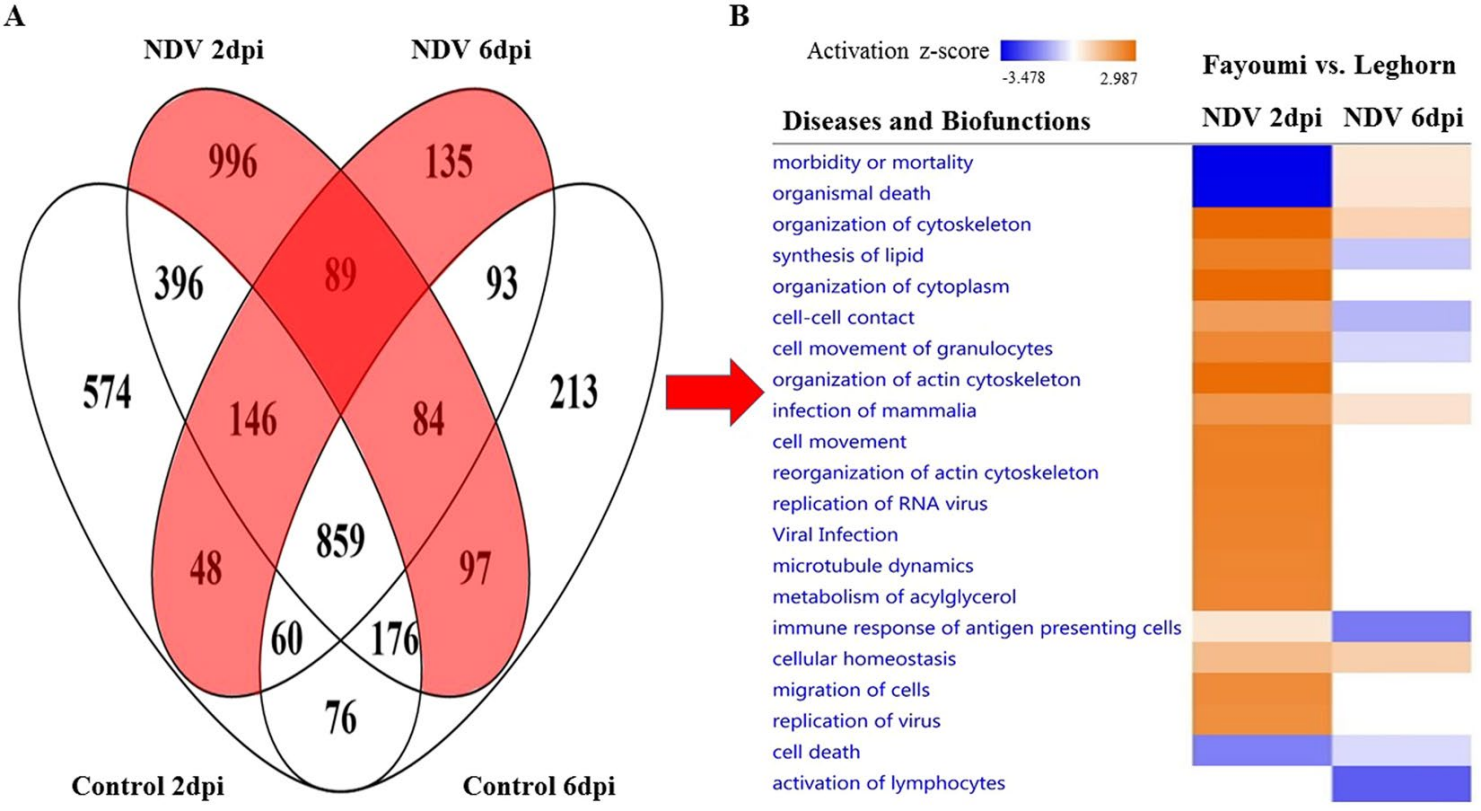
Differences of significant DEGs and biofunctions between Fayoumis and Leghorns are altered by NDV from 2 to 6 dpi. (**A**) Venn diagram showing unique DEGs for Fayoumi vs. Leghorn induced by NDV (red color) at different time points. (**B**) Heat map showing Diseases and Biofunctions predicted by IPA based on the DEGs. Prediction of lower or higher activity of a disease or biofunction in Fayoumis than Leghorns is calculated as negative or positive z-score and colored in blue or orange, respectively, in the heat map. The intensity of the color in the heat map based on |z-score| indicates degree of the predicted difference.

For the contrast between lines, most top DEGs (FDR <0.05) with highest fold changes are unannotated genes without known functions, but our study highlighted their involvement in some respect of host resistance to NDV (Supplementary Table S2). More DEGs with FDR less than 0.05 were always found at 2 dpi than 6 dpi, regardless of whether the birds were NDV challenged or not. NDV challenge resulted in more DEGs at 2 dpi but fewer DEGs at 6 dpi (Fig. 3B, Supplementary Table S2). A total of 859 DEGs were shared between 2 dpi and 6 dpi and were not affected by NDV challenge (Fig. 4A), accounting for a large proportion (30–57%) of the total number of DEGs in each group. There were also 1,266 and 418 DEGs for the Fayoumi vs. Leghorn contrast that were induced by NDV challenge at 2 and 6 dpi, respectively (Fig. 4A).

### Regulatory pathways predicted by Ingenuity Pathway Analysis (IPA)

When we input the whole gene list for each contrast into IPA and set the threshold at FDR <0.05, IPA predicted significant pathways (p < 0.05) based on the overlap of observed and predicted DEGs and also significant activation (z-score >2) or inhibition (z-score <−2) of certain pathway based on overlap of observed and predicted expression change of each DEG in the pathway. Among the top five pathways for NDV-infected vs. non-infected Leghorn chickens at 2 dpi, IPA predicted activation of two pathways regulating cell apoptosis induced by NDV, including “retinoic acid mediated apoptosis signaling” and “death receptor signaling”, due to upregulation of apoptotic peptidase activating factor 1 (*APAF1*), poly (ADP-ribose) polymerase family member 9 (*PARP9*), 12 (*PARP12*) and 13 (*PARP13*) (Table 3). In addition, three pathways regulating immune response were also significantly changed by NDV challenge: role of protein kinase R (PKR) in interferon induction and antiviral response, interferon signaling, and complement system (Table 3).

**Table 3:** Top significant pathways (p < 0.05) predicted by IPA for NDV vs. Control by line and day post infection (2 dpi or 6 dpi).

Group	Pathways	DEGs in the Pathway	P-value	Ratios
Leghorn 2 dpi	Retinoic acid mediated apoptosis signaling*	*APAF1, PARP9, PARP12, PARP13*	2.8E-05	4/61
	Death receptor signaling*	*APAF1, PARP9, PARP12, PARP13*	1.4E-04	4/92
	Role of PKR in interferon induction and antiviral response	*APAF1, EIF2AK2, STAT1*	2.1E-04	3/40
	Interferon signaling	*STAT1, TAP1*	4.8E-03	2/36
	Complement system	*C1S, SERPING1*	5.1E-03	2/37
Fayoumi 2 dpi	Role of lipids/lipid rafts in the pathogenesis of influenza	*RSAD2*	0.014	1/22
	Complement system	*C1S*	0.024	1/37
	Role of PKR in interferon induction and antiviral response	*EIF2AK2*	0.026	1/40
	Role of RIG1-like receptors in antiviral innate immunity	*TRIM25*	0.028	1/43
	Retinoic acid mediated apoptosis signaling	*PARP9*	0.040	1/61
Leghorn 6 dpi	Granzyme A signaling	*GZMA*	0.011	1/20
	Role of lipids/lipid rafts in the pathogenesis of influenza	*RSAD2*	0.012	1/22

Note: Pathways labeled with * were predicted to be significantly upregulated (z-score >2). All differentially expressed genes (DEGs) were significantly upregulated (False discovery rate <0.05). No pathway was predicted for Fayoumi at 6 dpi. Ratios = (Number of DEGs in a pathways)/(Total number of genes in the pathway).

**Figure 5:**
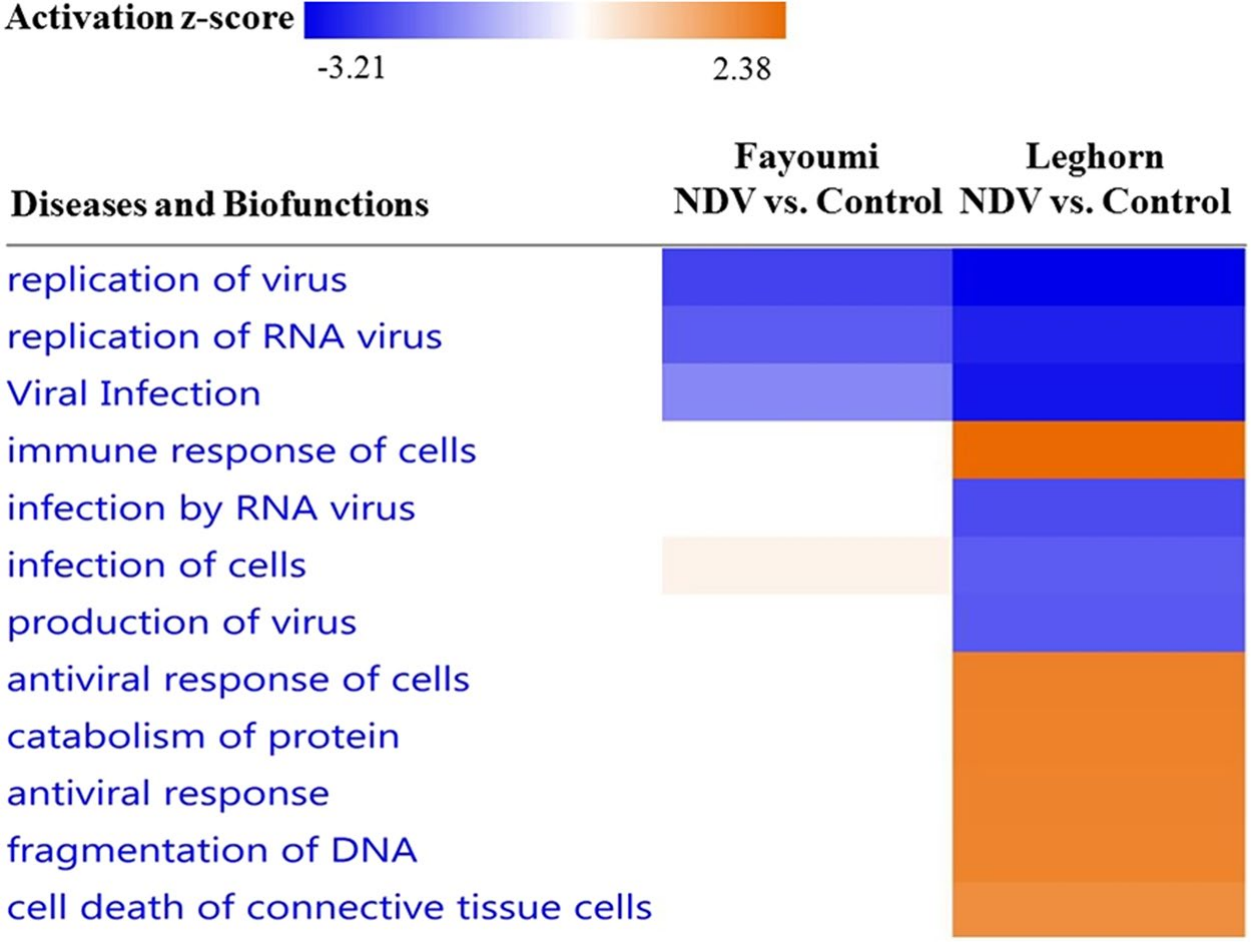
Comparison between two chicken lines on Diseases and Biofunctions predicted by IPA for NDV vs. Control at 2 dpi. Prediction of activation or inhibition of a disease or biofunction by NDV is calculated as negative or positive z-score and colored in blue or orange, respectively, in the heat map. The intensity of the color in the heat map based on |z-score| indicates robustness of the prediction.

In Fayoumi chickens at 2 dpi, “Retinoic acid mediated apoptosis signaling”, “role of PKR in interferon induction and antiviral response”, and “Complement system” were also among the top five significant pathways altered by NDV challenge. In addition, “Role of lipids/lipid rafts in the pathogenesis of influenza”, and “Role of RIG1-like receptors in antiviral innate immunity” were ranked as top significant pathways as well (Table 3). Comparing response to NDV between the two lines at 2 dpi, we found an interesting pattern of predicted changes in the disease and biofunctions section in IPA. In Leghorn chickens, IPA predicted not only inhibited viral replication and infection but also activated antiviral response of immune cells and activation of cell apoptosis activities in response to NDV challenge such as protein catabolism and DNA fragmentation. In contrast, in Fayoumi chickens, NDV seemed to only induce inhibitory regulation of viral replication and infection (Fig. 5).

In Leghorn chickens at 6 dpi, only two significant pathways were predicted to change in response to NDV. Similar to Fayoumi chickens at 2 dpi, the role of lipids/lipid rafts in the pathogenesis of influenza was predicted due to upregulation of *RSAD2*. In addition, “Granzyme A signaling” was predicted as a unique pathway because of unique upregulation of granzyme A (*GZMA*) in Leghorn chickens at 6 dpi (Table 3).

For contrasts between lines, many significant pathways were identified for each contrast within each treatment at each time point because there were many DEGs, but almost all the top five pathways were related to immune function and cell survival, even between the control birds. As shown in Table 4, some pathways were highly significant regardless of the treatment, such as “Production of nitric oxide and reactive oxygen species in macrophages”, and “IL-8 signaling” at 2 dpi, and “coagulation system” at 6 dpi. However, most pathways were unique to each comparison, and even the shared pathways such as “coagulation system” showed different DEGs, indicating large effects of time and NDV treatment on differential expression between the two lines. Despite the different pathways for each set of comparisons, some important genes had universal involvement in several pathways, such as genes in phosphoinositide 3-kinase (PI3K) complex, including the PI3K family and the fibroblast growth factor receptor (FGFR) family. When the chickens were challenged by NDV, several genes in the Eukaryotic Translation Initiation Factor (EIF) family, such as *EIF2S3*, *EIF3D*, *EIF3F*, *EIF3L*, *EIF3M*, and EIF4E binding protein 1 (*EIF4EBP1*), seemed to become key contributors to differential response between Fayoumi and Leghorn at 2 dpi (Table 4). Interestingly, all these genes, except *EIF3D*, were only significantly higher in expression in the challenged birds of Fayoumis than Leghorns at 2 dpi, indicating their possible important role in regulating early resistance to NDV.

**Table 4:** Top five pathways (p < 0.01) predicted by IPA for Fayoumi vs. Leghorn in different treatment groups.

Group	Pathways	Important DEGs contributing to prediction	Ratios
Control 2 dpi	IL-8 signaling	***FGFR(1/3), PIK3C(G/2 G),GNG(2/10)***	29/197
	Production of nitric oxide and reactive oxygen species in macrophages	***PIK3C(G/2 G), FGFR(1/3)*** *, NCF(1/2/4)*	25/193
	STAT3 pathway	***PDGFR(A/B), FLT(1/4), FGFR(1/3/L1)***	17/73
	Sumoylation pathway	***FAS, FASLG, SENP1, RHOJ*** *, CBX4*	15/96
	Sphingosine-1-phosphate Signaling	***FGFR(1/3), PDGFA, PIK3C(G/2 G)***	17/123
NDV 2 dpi	mTOR signaling	*ATG13, EIF3D/F/L/M, EIF4EBP1*	35/199
	Regulation of eIF4 and p70S6K signaling	*PABPC1, EIF3(D/F/L/M), EIF4EBP1*	29/157
	EIF2 signaling	*PABPC1, EIF3(D/F/L/M), EIF2S3*	**36/221**
	IL-8 signaling	***FGFR(2/3), PIK3C(G/2 G), RHOB***	29/197
	Production of nitric oxide and reactive oxygen species in macrophages	***STAT1, PIK3C(G/2 G), FGFR(2/3)***	28/193
Control 6 dpi	Coagulation system	*F7, F10, FGB*, ***SRPINF2, BDKRB1***	9/35
	Leukocyte extravasation signaling	***ACT(A1/A2/G2), FGFR(2/3), PIK3C2G***	23/210
	Th2 pathway	***PIK3C2G, CD(4/28), FGFR(2/3), DLL1***	16/150
	Paxilin signaling	***ACT(A1/A2/G2), FGFR(2/3), PIK3C2G*** *,*	13/113
	Acute myeloid leukemia signaling	***PIK3C2G, KLB, FGFR(2/3), KIT***	11/91
NDV 6 dpi	Complement system	***C1S, C1R, C8B, CFD, MBL2***	5/37
	eNOS signaling	***VEGFD, FGFR3, PIK3C2G*** *, CNGA3*	11/155
	PCP pathway	***WNT5A, RSPO3, CELSR1, FZD(3/5)***	6/63
	Oncostatin M signaling	***MMP(1/3), PLAU***	4/34
	Coagulation system	***SRPINF2, F13A1, PLAU*** *, TFPI*	4/35

Note: Bold italic and italic text respectively indicates lower and higher expression of differentially expressed genes (DEGs) in Fayoumi compared to Leghorn. Genes within the same family are labeled with the member or subunit names in the brackets. Ratios = (Number of DEGs in a pathways)/(Total number of genes in the pathway).

The induced differential expression of EIF family members by NDV between the two lines suggests that differences between the two lines unique to the challenged birds may be more indicative of resistance and susceptibility than the other DEGs. Therefore, we entered the DEGs between lines that are unique to challenged chickens (Fig. 4A) for each time point into IPA to compare prediction of diseases and biofunctions between 2 and 6 dpi. With DEGs between lines induced by NDV at 2 dpi, IPA predicted lower morbidity or mortality, higher lipid metabolism activity, higher activity of cytoskeleton and cytoplasm organization, more active movement of granulocytes, and more dynamic viral infection and immune response, in Fayoumis than in Leghorns (Fig. 4B). However, prediction of most of these differences became weak or vanished (z-score <2) at 6 dpi, and the unique DEGs induced by NDV between the two lines seemed to contribute to higher morbidity and mortality, lower immune response and activation of lymphocytes, and less dynamic movement of granulocytes and immune cell movement activity, in Fayoumis than in Leghorns (Fig. 4B).

### Co-expression analysis with WGCNA

**Figure 6:**
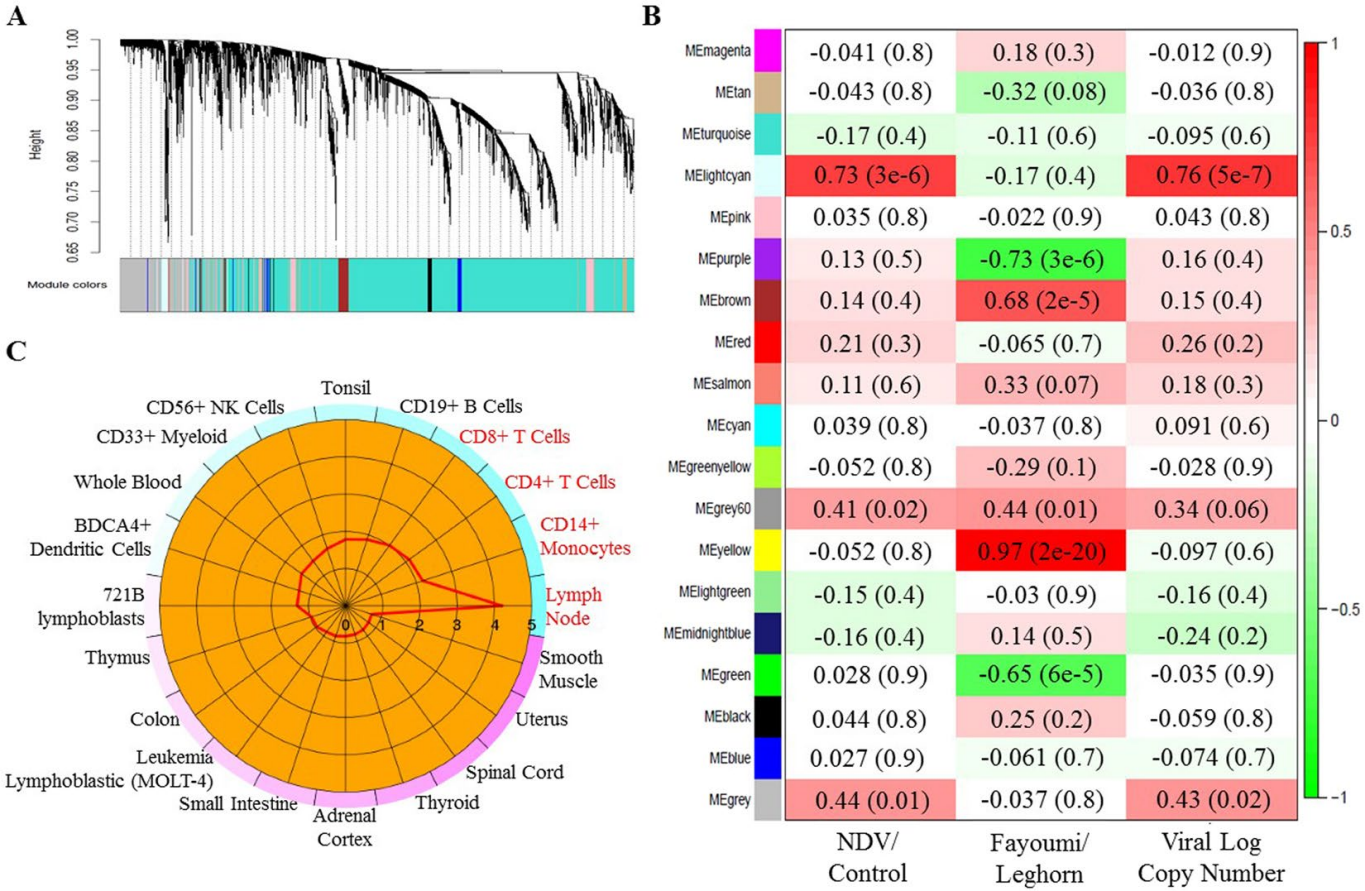
WGCNA co-expression analysis revealed important modules with strong correlations with treatment, line, and viral load in tears. (**A**) Cluster dendrogram showing 19 modules of genes identified by WGCNA with similar expression patterns across different group. (**B**) Heatmap showing correlation of each module eigengene with treatment, line, and log_10_ (viral copy number in lachrymal fluid). Red and green colors respectively represent positive and negative correlations. Intensity of the color is determined by the absolute value of the correlation coefficient, which is also labeled in each box with p-value in parenthesis. (**C**) Genes in lightcyan module showed significant enrichment (enrichment score >2) of several immune cell types (colored in red) in CTen analysis. Red line in the circle indicates enrichment score calculated as −log_10_ (Benjamini-Hochberg adjusted P value). The color around the circle changes from blue to purple as the enrichment score decreases.

Although differential expression analysis identified significant DEGs between groups, many genes with similar expression pattern among different groups were filtered out due to insignificance. Therefore, we normalized the read counts from HTseq using the DEseq. 2 package, and performed weighted gene co-expression network analysis (WGNCA) to cluster genes into different modules based on their expression patterns. A total of 19 modules containing 33 to 3,694 genes were identified in WGCNA (Fig. 6A). The lightcyan module showed significant positive correlation with both NDV challenge (r = 0.73) and NDV copy number in lachrymal fluid (r = 0.76) (Fig. 6B), indicating that expression of genes in this module were all upregulated by NDV challenge and the extent of their upregulation may be related to the NDV viral load in the body. When these genes were entered into the gene ontology (GO) enrichment analysis, “negative regulation of viral replication”, and “response to virus” were listed as significant (p < 0.05) gene ontology (GO) terms (Table 5). Interestingly, *RSAD2*, *Mx*, 2′–5′-oligoadenylate synthetase like (*OASL*) and eukaryotic translation initiation factor 2 alpha kinase 2 (*EIF2AK2*), which are DEGs for NDV vs. control, are also in the lightcyan module and involved in these GO terms. From the genes in lightcyan module, cell type enrichment (CTen) analysis predicted enrichment of CD14+, CD4+ and CD8+ cells in response to NDV challenge (Fig. 6C), indicating that these cells may play significant roles in defense against NDV.

**Table 5:** Gene ontology annotation results for genes in modules with a strong correlation (p < 0.05) with treatment or genetic line.

Module	Gene No.	Biological Process	Accession No.	P-value	Represented Genes
Lightcyan	61	Negative regulation of viral genome replication	GO:0045071	0.009	*EIF2AK2, OASL, RSAD2, STAT1*
		Response to virus	GO:0009615	0.006	*CCL19, EIF2AK2, Mx, OASL, RSAD2*
Brown	1383	Translation	GO:0006412	2.4E-16	*EIF3(I/J), EIF4E(3/BP1), RPL(3/4/6/8/9/12/13/19/21/23/27/31/32/37/39), MRPL(10/20/22/44/47/51), RPS(2/3/7/14/16/21/24/28/29)*
		Mitochondrial transport	GO:0006839	0.022	*BCL2L1, BNIP3, MPC(1/2), MTX2, SLC25A(4/17/21/43)*
		Ribosome biogenesis	GO:0042254	0.016	*RPL(6/10 A/12/23 A), RPS(10/14/16/21/24/28), UTP(11 L/20)*
		Cellular component organization	GO:0016043	0.006	*CHCHD(4/10), COX(17/18/19), EIF3(I/J), KCTD(2/3), RPS(3/10/14/28), RPL(12/23), SLC25A(4/33), SNX(3/5)*
Yellow	1303	Cell communication	GO:0007154	0.006	*CD (4/28/151/180), GPR (27/39/144/149/157/162)*
		Cellular response to stimulus	GO:0051716	0.002	*CD (4/28/151/180), EIF2AK1, FAS, GPR (27/39/144/149/157/162), PIK3C2G, PLA2R1, TNFRSF(18/21)*
		Cellular nitrogen compound biosynthetic process	GO:0044271	0.025	*CHDH, CMPK, CPEB1, GUCY(1B2/2 C), HOXA7, NPPA, PAPSS1, PHOX2B, RX1, SUPT3H, TERT, THRB, UPP1*
Green	1259	Organic substance transport	GO:0071702	0.037	*CD40LG, EIF5A2, SLC2A(6/9), SLC16A(1/7/10), SLC27A(4/6), SLC(15A4/17A5/18A2/22A3/26A5/29A4/35A3)*

Note: Genes within the same family are labeled with the member or subunit names in the brackets.

There are also two modules [brown (r = 0.68) and yellow (r = 0.97)] that showed significant positive correlations and two modules [purple (r = −0.73) and green (r = −0.65)] that showed negative correlation with chicken lines in WGCNA. Because we designated Fayoumis as 1and Leghorns as 0, the positive and negative correlation would respectively mean higher or lower expression in Fayoumis than Leghorns. GO term analysis indicated that genes in the brown module are significantly (p < 0.05) enriched in biological processes including translation, ribosome biogenesis, mitochondrial transport, and cellular component organization (Table 5). Many genes encoding components in 40S (RPS) and 60S (RPL) subunit of ribosomes, several genes encoding 39S subunit of mitochondrial ribosomal (MRPL) proteins, and several mitochondrial carrier subfamily of solute carrier protein (SLC25) genes all contribute to enrichment of these GO terms. Genes in the yellow module are significantly (p < 0.05) enriched in cell communication, cellular response to stimulus, and cellular nitrogen compound biosynthetic process. Genes encoding several cluster of differentiation (CD) antigens and G protein-coupled receptors (GPR), and several aforementioned important genes in IPA (Table 4) including *PIK3C2G* and *FAS*, are major genes contributing to enrichment of the GO terms. Genes in the green module were significantly enriched in organic substance transport. Among them, genes in solute carrier family (SLC) represent a large proportion (Table 5). Interestingly, there are EIF family members in all three modules, indicating that EIF family may play an important role in resistance to NDV in Fayoumi chickens.

## Discussion

To improve resistance to NDV in chickens, we need to understand how gene expression in response to this pathogen is affected by genetic resistance. Fayoumi chicken line, as a highly inbred line with outstanding resistance to various pathogens, serves as an excellent model for this research objective. Following comparison of gene expression in tracheal epithelium between Fayoumi and a disease susceptible Leghorn line in an NDV challenge study^13^, we further compared gene expression in spleen in the same study. Similar to the previous finding in trachea, the number of DEGs induced by NDV in spleen decreased from 2 to 6 dpi in both chicken lines. The fewer DEGs in spleen compared with those in trachea in the NDV vs. control contrast indicate moderated response to NDV in this tissue. However, the difference of DEG number at 2 dpi between the two lines became larger in spleen than in trachea. The number of DEGs induced by NDV in Leghorns was only 1.1 times higher than that in Fayoumis at 2 dpi in trachea, but became 5.3 times higher in spleen, indicating a large effect of differential genetic resistance between the two lines on the early immune defense to the virus. Certainly, the difference in genetic resistance cannot be contributed only by the spleen, as even in trachea at 2 dpi, Fayoumis have shown significantly lower NDV transcript number than Leghorns^13^. And the lesser effect of NDV challenge on splenic gene expression in Fayoumis compared to Leghorns is perhaps due to faster viral clearing during viral circulation from trachea to spleen in this resistant line.

Among the 18 DEGs induced by NDV in spleen of Fayoumis at 2 dpi, 15 of them were also found in Leghorn at 2 dpi (Table 2), suggesting a common basic mechanism of immune regulation in spleen in both lines in early response to NDV. Large-fold upregulation of *Mx*, *IFIT5, OASL* and *EIF2AK2* in spleen of Leghorn chickens challenged by a virulent strain NDV-CA02 at 24 and 48 hour post infection^15^ not only confirmed the involvement of these genes in regulation of splenic response to NDV but also indicated positive correlation between the extent of expression change and virulence of virus. Expressions of these genes seem also positively related to NDV load in the lachrymal fluid as they were in lightcyan module in WGCNA (Table 5), indicating their important roles in host defense to NDV. Due to difference of reference genome version, only 10 of the 15 DEGs mentioned above were found in both spleen and trachea. Examining the function of these genes, we found all of these genes are directly or indirectly induced by interferon. For example, *Mx*, *OASL* and *EIF2AK2* showed continuously elevated abundance and similar expression profile in spleen of Leghorn chickens from 3 to 9 hours after injection of chicken IFN-α^16^. These three genes are known for their functions to respectively trap virus, cleave viral RNA, and inhibit translation of viral protein^17^. Therefore, upregulation of these genes is an expected observation in response to viral challenge and thus validated the spleen and tracheal epithelium as appropriate tissues to study gene expression response and genetic resistance to NDV.

Generally, the aforementioned genes are mainly involved in innate immunity, which is the first line of host defense against virus, and their upregulation commonly leads to negative viral replication, as indicated by IPA analysis with DEGs for NDV-challenged vs. control contrast at 2 dpi (Fig. 5) and GO term analysis with genes in the lightcyan model in WGCNA (Table 5). The prediction of lymph node and immune cell enrichment with genes in lightcyan model (Fig. 6C) also indicate a conserved cellular response to NDV between the two chicken lines and a strong immune cell enrichment induced by challenge at both 2 and 6 dpi. Interestingly, with DEGs for NDV vs. control, significant enrichment of lymph node, CD14+ monocytes, CD4+ and CD8+ T cells was also predicted in the trachea of both Fayoumis and Leghorns at both 2 and 6 dpi^13^, indicating that a core cellular response to NDV may exist across not only different chicken lines and time points but also different immune tissues. Because the prediction of the CTen analysis is based on human and mouse data, lymph node, which does not exist in chicken was predicted. But 3–10% increase of CD4+ and CD8+ T cells in peripheral blood mononuclear cells has been found in chickens receiving a booster dose of inactivated NDV vaccines prepared from either lentogenic or velogenic strain^18^, indicating enhanced T cell immune response and increased lymphocyte transformation after a booster vaccine. CD4+ T cells are T helper cells that regulate immune response by secreting T cell cytokines, while CD8+ T cells are cytotoxic T cells that recognize and destroy the infected cells, and memorize the antigen during differentiation from naive cells to effector cells and memory cells^19^. The spleen has more CD8+ T cells than CD4+ T cells^19^. It has been reported that CD4+ T cells are critical to promote clonal expansion^20^, sustain virus-specific activity of CD8+ T cells during chronic viral infection^21^, and develop CD8+ T cell memory^22^. Therefore, both CD4+ and CD8+ T cells may be important for immune response to NDV.

Despite the common response to NDV of these two chicken lines, there are many differences between the two lines as indicated by the huge number of DEGs (Fig. 4B). These differences are of interest as the potential basis for resistance versus susceptibility in response to NDV. Also, according to IPA, there is strong inhibition of infection of host cells, and strong activation of immune and antiviral response, protein catabolism, DNA fragmentation, and cell death in response to NDV challenge only in Leghorn chickens at 2 dpi (Fig. 5), suggesting possible apoptosis induction of infected cells in the spleen of Leghorn chickens, which agrees well with the numerous apoptotic macrophages and lymphoid cells observed by Anis *et al*. in spleen of white Leghorn chickens at 2 dpi after inoculation of virulent 9a5b NDV^23^. This result also agrees with the activation of “Retinoic acid mediated apoptosis signaling” and “Death receptor signaling” predicted by IPA (Table 3), because both pathways are involved in the regulation of T-lymphocyte apoptosis^24,25^. Interestingly, both extrinsic apoptotic pathways involve four unique DEGs (*APAF1*, *PARP9*, *PARP12* and *PARP13*) induced by NDV at 2 dpi in Leghorn chickens (Table 3). In both pathways, APAF1 is required for formation of apoptosome complexes which activate caspase-3 or 7^26,27^. Poly (ADP-ribose) polymerase (PARP), comprised of PARP9, 12 and 13, contributes to cell death by depleting cells of nicotinamide adenine dinucleotide (NAD) and adenosine triphosphate (ATP)^28,29^. However, because binding of PARP to DNA strand breaks protect genome integrity, subsequent cleavage of PARP by caspase-3 or 7 is necessary for DNA fragmentation during cell apoptosis^30^. Therefore, upregulation of *APAF1*, *PARP9*, *PARP12* and *PARP13* may lead to higher NDV-triggered apoptosis activity.

In addition to higher cell apoptosis activity triggered by NDV, Leghorn chickens seem to show higher activity in several immune-related pathways even without NDV challenge (Table 4). Interestingly, most of these pathways involve several genes in phosphatidylinositide 3-kinase (PI3K) complex, indicating the great contribution of PI3K to the difference between these two chicken lines. PI3K has been reported to be critical in gate-keeping system to prevent excessive Th1 polarization and innate immune response^31^. Therefore, higher expression of genes in PI3K complex in Leghorn chickens may be related to their higher susceptibility to infectious diseases. Another important gene family that may contribute to the different resistance between the two chicken lines is the EIF family, because several genes in this family showed uniquely higher expression in response to NDV in Fayoumi than Leghorn at 2 dpi and promote mRNA translation in three top pathways (Table 4). Among these pathways, “mTOR signaling” has been known to regulate CD8 T cell differentiation^32^ and toll-like receptor-mediated induction of IFN-I in plasmacytoid dendritic cells, and to be inhibited during autophagy-mediated cell death after viral infection^33,34^. “Regulation of eIF4 and p70S6K signaling” pathway participates translational regulation in apoptosis in response to physiological stress^35^. “EIF2 signaling” is also predicted to have higher activity in trachea of Fayoumis compared to Leghorns at 2 dpi^13^, and it has been known to inhibit viral replication and regulate proinflammatory cytokine expression^36^. Interestingly, all three pathways were reported to show decreased methylation in chickens immunized with infectious laryngotracheitis vaccine compared to the unimmunized ones^37^. Therefore, upregulation of these pathways may be vitally important for enhanced disease resistance in Fayoumi chickens. All three pathways are involved in translation regulation, and thus higher expression of EIF family members indicate higher translational activity in Fayoumis compared to Leghorns. This result is also supported by GO enrichment in “Translation” and “Ribosome biogenesis” by genes in brown module in WGCNA (Table 5) which showed higher expression in Fayoumi chickens (Fig. 6C). Many genes encoding ribosome proteins and mitochondrial ribosome proteins are contained in this module, indicating their relatively higher expression in Fayoumi chickens although they may not be significant.

The enhanced translation activity at 2 dpi in Fayoumis seems to contribute to prediction of lower organismal morbidity and mortality in Fayoumi chickens, which may be related to higher activity of lipid synthesis, organization of actin cytoskeleton, microtubule dynamics, cell-cell contact, cell movement, and immune response of antigen presenting cells (Fig. 4B). It has been shown that lipogenesis was inhibited and lipolysis was strengthened in chicken blood within 3 days after infection of NDV^38^. Therefore, higher lipid synthesis activity may related to higher resistance to NDV. Higher actin cytoskeleton organization agrees well with the enrichment of genes in the WGCNA brown module in cellular component organization (Table 5). Activation of organization of actin cytoskeleton by NDV was also predicted to be stronger in the trachea of Fayoumis than that of Leghorns^13^. Actin cytoskeleton, as an integral component of lymphocyte activation, plays a critical role not only in T lymphocyte migration toward antigen in the body, but also in formation of the immunological synapse between antigen presenting cells and T lymphocytes^39^. In addition, it also regulates T cell signaling by forming a signaling complex, and recruiting or stabilizing membrane domains implicated in T cell activation^40^. Therefore, higher activity of actin cytoskeleton organization also corresponds to higher activity of microtubule dynamics, cell communication, cell movement and immune response to antigen presenting cells, and may be a major mechanism for higher resistance of Fayoumi chickens to infectious diseases. This hypothesis is supported by enrichment of actin filament-based movement in lung by the upregulated genes induced by avian influenza virus in Fayoumi chickens^12^, and also by down-regulation of several genes encoding actin proteins by NDV in avian cells^41^.

The higher activity of immune cell movement and communication in Fayoumi chickens is also supported by inclusion of genes encoding CD antigens and GPRs in the yellow module in WGCNA (Table 5), because genes in yellow module showed higher expression in Fayoumi and CD antigens and GPRs are enriched in “cell communication and cellular response to stimulus” in the GO term analysis. In addition, enrichment of genes in yellow module in the GO term “cellular nitrogen compound biosynthetic process” also corresponds to the prediction of “production of nitric oxide and reactive oxygen species in macrophage” and “eNOS signaling” pathways with DEGs for Fayoumi vs. Leghorn at 2 dpi in IPA, indicating a potential influence of nitric oxide (NO) on different disease resistance between the two chicken lines. NO is a nitrogen compound that has defense function against various viral infection^42^, and it has been reported to be induced by NDV in chicken heterophils and by *Eimeria tenella* in macrophages from chicken spleen^43,44^. Moreover, production of NO induced by *E.coli* lipopolysaccharide (LPS) or *E. tenella* sporozoites in spleen lymphocyte culture within 8 days post infection was higher in SC (B2B2) chickens which are relative resistant to coccidiosis, but lower in TK (B15B21) chickens which are susceptible to coccidiosis^44^. Production of NO induced by LPS in bone marrow derived dendritic cells at 1 day post treatment was also higher in Fayoumis than in Leghorns^45^. Therefore, higher resistance to NDV of Fayoumis than Leghorns may be also related to their differential NO production in immune cells.

In addition to disease resistance, Fayoumi chickens also have relatively higher tolerance to heat stress than many other chicken lines due to natural selection by the high-temperature climate in its origin region. Comparing with the result of a previous study which investigated gene expression in spleen under heat stress condition^46^, we found most significant pathways are different between these two studies. Therefore, different mechanisms may be involved in regulating resistance to the two stressors. However, the “coagulation system” pathway, which is a significant pathway between Fayoumis and Leghorns regardless of the treatment in this study, is also among the significant pathways altered by heat stress in Fayoumi. Clotting proteins in the blood are known to be consumed in the disseminated intravascular coagulation (DIC) caused by heat stroke in human, which can eventually lead to tissue damage^47^. Integrally related to innate immune system, coagulation is also activated by inflammation to prevent invasion of pathogens. But dysregulated coagulation system can also form positive feedback loop with inflammation which eventually leads to DIC and septic shock^48^. Therefore, properly regulated coagulation system is important for resistance to both heat stress and viral infection, and the Fayoumi chickens may be better protected by their coagulation system. This may be also the reason why LPS induced few DEGs but LPS+ heat stress induced many more DEGs in spleen of Fayoumi chickens^46^. Thus it is also possible that response to NDV in these chickens would also be altered by heat stress. However, further studies are still needed to validate this speculation. In summary, we identified the DEGs in response to NDV in both Fayoumis and Leghorns, and found significant pathways and GO terms involving these genes in spleen. Through comparison between these two lines, we found genes and pathways that putatively contribute to the resistance of Fayoumi to NDV. To identify an ideal target and a feasible strategy for genetically improving resistance to NDV in chickens, further comparison of gene expression in other immune tissues and performing functional studies on the identified genes and pathways is warranted.

## Methods

### Animals and Experimental Design

This study was approved by the Iowa State University Institutional Animal Care and Use Committee (IACUC log number 1–13–7490-G) and has been described previously^13^. All experiments were performed in accordance with the relevant guidelines and regulations. Fayoumi (n = 40) and Leghorn chickens (n = 49) of the same age were raised in normal conditions after hatch with ad libitum access to feed and water. At 21 days posthatch (dph), each chicken line was randomly divided into two groups. One group (n = 49) was challenged with the LaSota lentogenic strain of NDV (200 μl of 10^7^ EID_50_%) through nasal and ocular inoculation routes (50 μl into each eye and nostril), while the control group (n = 40) was treated with 200 μl phosphate-buffered saline (PBS) through the same routes. Lachrymal fluid from the surface of the eye of each chicken was collected pre-challenge (n = 89) and at 2 (n = 89) and 6 (n = 62) days post-inoculation (dpi) with a pipette after placing granular sodium chloride on the eye. Viral RNA in lachrymal fluid was isolated using the MagMAX^TM^-96 viral RNA Isolation Kit (Life Technologies, Carlsbad, CA) and quantified with the LSI VetMAX^TM^ Newcastle Disease Virus Real-Time PCR Kit (Life Technologies) on the MJ Research Opticon2 qPCR machine (Bio-Rad, Hercules, CA). We euthanized 24 and 30 chickens with sodium pentobarbital solution respectively at 2 and 6 dpi. Four chickens were randomly selected for RNA-seq within each treatment group in each line at each time point and their NDV viral loads in lachrymal fluid were compared through two-way ANOVA and Tukey HSD posthoc test in JMP13.2. Spleen tissues were collected and placed into RNA*later* solution (ThermoFisher Scientific, Waltham, MA) for short-term storage and transferred to a −80 °C freezer.

### Total RNA Isolation

RNA samples were isolated from spleens using Ambion RNAqueous Total RNA Isolation Kit (Thermo Fisher Scientific) following the manufacturer’s protocol. Proper quantity and quality of the RNA samples were ensured respectively through assessment by NanoDrop ND-1000 UV-vis spectrophotometer (Thermo Fisher Scientific) and RNA 6000 Nano kit in Agilent 2100 Bioanalyzer (Agilent Technology, Santa Clara, CA). The RNA integrity number for each sample was above 8.

### cDNA Library Construction and Sequencing

The transcriptome library of each sample was constructed with 0.5 µg total RNA using the Illumina TruSeq RNA sample preparation kit (Illumina Inc., San Diego, CA) following the low sample protocol in the TruSeq RNA sample preparation v2 guide (Part #15026495, March 2014). The proper length of cDNA in each library was ensured by running the DNA 1000 assay on the Agilent 2100 Bioanalyzer. Libraries from 2 and 6 dpi were independently constructed and sequenced at different times. Two libraries randomly selected from four samples within each of the four line by treatment combinations were multiplexed and pooled into one lane for sequencing, so there were 2 sequencing lanes for each time point. Sequences of 100-bp single-end reads in each lane were obtained using the HiSeq 2500 Sequencing System (Illumina) at the Iowa State University (ISU) DNA facility. Sequencing of one control sample in Leghorn chickens at 2 dpi failed due to contamination and was excluded from further analysis.

### Sequence Reads Quality Control, Mapping and Counting

After quality assessment of raw reads using FastQC (version 0.10.1), adapter sequences and sequences of low quality (Sanger base quality <20) were trimmed using the FASTX-Toolkit in Cyverse (https://de.cyverse.org/de). The filtered reads of each sample were then aligned to the *Gallus gallus* Galgal 5.0 reference genome (assembly GCA_000002315.3) from Ensembl using TopHat2 (version 2.0.9) with default parameters. The mapped reads were subsequently counted using HTSeq (version 0.5.4), using default parameters and the *Gallus gallus* Galgal5.0 GTF file from Ensembl.

### Differential Expression, Pathway, and Co-expression Analyses

Because the library construction and sequencing were done separately for each time point, differential expression analyses were performed with data at 2 dpi and 6 dpi separately. Differential expression analysis was performed for comparisons between lines receiving the same treatment and between treatments within the same line using EdgeR software (version 3.12.1) in R (version 3.2.3). The trimmed mean of M-value (TMM) method was used to normalize read counts. The generalized linear model (GLM) based on a negative binomial distribution was used for model fitting with main effects including line and treatment. The Benjamini-Hochberg method was used to control the false discovery rate (FDR). Principle component analysis (PCA) plots weres generated by the ggbiplot program in R. Differentially expressed genes (DEGs) with FDR <0.05 were included in the Ingenuity Pathway Analysis software (IPA, QIAGEN, Redwood City, CA), and pathways or functions with |z-score| >2 were considered to be activated or inhibited^49^.

With input of treatment (NDV: 1, Control 0), line (Fayoumi: 1, Leghorn: 0), and viral load in the lachrymal fluid at the time of tissue collection as covariates, we used the weighted gene co-expression network analysis (WGCNA, version 1.51) package in R to perform co-expression analysis in order to cluster highly correlated genes and find modules that were significantly correlated with treatment, viral load and line. In addition, genes in significant modules were imported into Gene Ontology Consortium (http://www.geneontology.org/) for GO terms analysis, and their human homologs were used for cell type enrichment (CTen) analysis (http://www.influenza-x.org/~jshoemaker/cten/).

### Fluidigm Biomark validation of DEGs

To validate RNA-seq results, Biomark Dynamic assay (Fluidigm, South San Francisco, CA) was used to measure gene expression in the same 31 RNA samples as used in the RNA-seq study. Based on their log_2_FC in RNA-seq analysis, we selected 40 genes that cover the full range of log_2_FC in the comparisons between treatments or lines. In addition, the functional importance of each gene in immune defense was considered for selection. Primers of these genes are shown in Supplementary Table S3. Primers of *AVD*, *CMPK2*, *MOV10*, *Mx* and *ZNFX1*^13^; and *SIK1* and *GAPDH*^50^; and *IL1B*^46^ are from previous studies. The other primers were designed by Fluidigm with amplicons around 100 bp and spanning multiple exons if possible. The geometric mean of C_t_ values of 3 housekeeping genes [glyceraldehyde-3-phosphate dehydrogenase (*GAPDH*), actin beta (*ACTB*) and hypoxanthine phosphoribosyltransferase 1 (*HPRT1*)] were used for normalization. For each sample, cDNA was prepared from 50 ng of RNA using Reverse Transcription Master Mix and preamplified at 12 cycles using Preamp Master Mix (Fluidigm) according to the manufacturer’s protocols. Gene expression detection in each sample was performed in duplicate using the Fluidigm 48.48 integrated fluidic circuit (IFC). Two IFCs were run in the BioMark HD (Fluidigm) Real-Time PCR system for the 2 dpi and 6 dpi samples, respectively, and data were analyzed using the Fluidigm Real-Time PCR Analysis software. Replicates of the same sample showing a shifted peak in melting curves were removed. Gene expression was compared between lines or treatments using the 2^(−ΔΔCt)^ method in Excel, and pairwise correlation and linear regression between Log_2_FC in Biomark assay and that in RNA-seq was calculated using the JMP Pro 12.0.1 software.

### Data availability statement

The raw sequencing data generated and analyzed in the current study are available in ArrayExpress repository in EMBL-EBI (http://www.ebi.ac.uk/arrayexpress/experiments/E-MTAB-5851/) with accession number E-MTAB-5851.

## Electronic supplementary material

Supplementary Tables

Supplementary Table S2
