# Supplementary material for: Transcriptome Analysis in Spleen Reveals Differential Regulation of Response to Newcastle Disease Virus in Two Chicken Lines
Source: Sci Rep. 2018 Jan 19;8:1278. doi: 10.1038/s41598-018-19754-8 (PMC5775430; doi:10.1038/s41598-018-19754-8)
Supplement: Supplementary file 1 — Supplementary Tables [file 41598_2018_19754_MOESM1_ESM.pdf]

Scientific Reports

Supplementary Information

Transcriptome Analysis in Spleen Reveals Differential Regulation of Response to Newcastle Disease Virus in Two Chicken Lines

Jibin Zhang, Michael G Kaiser, Melissa S Deist, Rodrigo A Gallardo, David A Bunn, Terra R Kelly, Jack C. M. Dekkers, Huaijun Zhou, Susan J Lamont

Supplementary Figures S1, Supplementary Method and Supplementary Tables S1-S2

Supplementary Table S1. Statistical summary of sequence reading, mapping and counting

Lines	Treatment	dpi	ID	Raw Reads	Mapped Reads	% of Mapped Reads	Detected Genes	Transcriptome Coverage
Fayoumi	Control	2	1557	10,568,042	9,522,994	90.1%	16,344	65.69%
Fayoumi	Control	2	1570	13,657,047	12,078,116	88.4%	17,121	68.81%
Fayoumi	Control	2	1583	13,105,618	11,062,981	84.4%	16,847	67.71%
Fayoumi	Control	2	1600	11,612,675	10,428,287	89.8%	16,831	67.65%
Fayoumi	NDV	2	1561	16,510,671	14,763,540	89.4%	17,186	69.07%
Fayoumi	NDV	2	1581	14,218,354	12,700,422	89.3%	17,148	68.92%
Fayoumi	NDV	2	1584	12,092,303	10,893,210	90.1%	16,648	66.91%
Fayoumi	NDV	2	1593	14,293,617	12,784,429	89.4%	17,060	68.57%
Leghorn	Control	2	1534	14,014,512	12,673,490	90.4%	17,138	68.88%
Leghorn	Control	2	1538	15,456,860	13,895,139	89.9%	17,306	69.56%
Leghorn	Control	2	1552	12,542,341	11,272,389	89.9%	16,828	67.63%
Leghorn	NDV	2	1509	13,776,343	12,296,605	89.3%	16,912	67.97%
Leghorn	NDV	2	1520	12,068,107	10,876,213	90.1%	17,081	68.65%
Leghorn	NDV	2	1537	8,783,347	8,047,582	91.6%	16,128	64.82%
Leghorn	NDV	2	1540	12,486,593	11,233,994	90.0%	17,059	68.56%
Fayoumi	Control	6	1573	14,069,177	12,553,006	89.2%	17,298	69.52%
Fayoumi	Control	6	1582	8,957,210	8,089,496	90.3%	16,501	66.32%
Fayoumi	Control	6	1591	12,299,470	10,965,813	89.2%	17,097	68.72%
Fayoumi	Control	6	1595	12,219,456	10,973,292	89.8%	17,011	68.37%
Fayoumi	NDV	6	1559	11,322,870	9,982,642	88.2%	17,000	68.33%
Fayoumi	NDV	6	1560	10,590,158	9,539,460	90.1%	16,478	66.23%
Fayoumi	NDV	6	1569	12,714,127	11,324,123	89.1%	16,952	68.13%
Fayoumi	NDV	6	1586	15,389,645	13,581,937	88.3%	17,120	68.81%
Leghorn	Control	6	1522	14,796,255	13,255,483	89.6%	17,362	69.78%
Leghorn	Control	6	1528	15,197,593	13,436,378	88.4%	17,267	69.40%
Leghorn	Control	6	1539	18,133,378	16,351,508	90.2%	17,521	70.42%
Leghorn	Control	6	1548	14,657,429	13,119,397	89.5%	17,493	70.31%
Leghorn	NDV	6	1503	14,667,085	13,214,819	90.1%	17,246	69.31%
Leghorn	NDV	6	1535	15,585,655	13,861,810	88.9%	16,973	68.22%
Leghorn	NDV	6	1546	18,183,922	16,300,927	89.6%	17,704	71.15%
Leghorn	NDV	6	1549	13,825,063	12,373,852	89.5%	16,974	68.22%

Note: The percentage of mapped reads was calculated as the number of mapped reads divided by the number of raw reads; Transcriptome coverage was calculated as the number of detected genes (genes with at least one mapped read) divided by the total number of genes in Galgal5.0 genome which is 24881.

Supplementary Table S2. Top differentially expressed genes (|Log_2FC|>1, FDR<0.05) in different contrasts between lines or treatments (.xlsx)

Supplementary Table S3. Primers used in Fluidigm Biomark q-PCR for the validations of RNA-seq data

Gene Symbol	GenBank Accession Number	Forward Primer 5’→3’	Reverse Primer 5’→3’
ABCB5	XM_015281858	CAACCAGCTCCAGGTGTAGATA	ACACAGCAAAGCCAATTCCC
ACE	NM_001167732	TGCACACCTGTGACATCTACA	GACTTAGAAGACCCAGCCTTCA
ACTB	NM_205518	CCGTGCTGTGTTCCCATCTA	AGCTGTCTTTCTGGCCCATAC
APOLD1	XM_004937676	GGGGAGGTCAGCCAGAAAC	TGCCTTGTGAAGGTGAGCAA
AVD	NM_205320	TTCACAGGCACCTACATCACA	GGTGTTTTGTGTCCCATGCA
C1S	NM_001030777	TGGGAACAGAGCTACCAACA	CTGCAAGAGTGCCTCCAAAA
CCL19	NM_001302168	TCCGAGAGAAGCTGGACAC	ACACACTTCTGCAGAGCCTA
CD180	XM_003642909	ACCCGCATAGTGGATGCA	CCAGGAATCTCCCTCAGTCC
CD8A	NM_205235	GAAGACGACGATGCAGGTGTA	TATGTGTCGGGTTGGCACAC
CHIR-B2	NM_001146140	GCCCTCATCCTGGGTTGG	TGGGGTGCAGCGACAG
CMPK2	XM_015284945	AGGCTGAACTGGAAGCTAACA	CTTGGCACGCAGGATTCAC
DDX60	XM_004940918.2	CCCCGAGTGCAACATTTCATA	CAACAATCACCGCAGACTCA
EPSTI1	XM_417033	CAGGAGGAAGAAGCCAGGATA	GAAGTCGGTCCAGAAAAGCA
FAM26F	XM_419776.3	GGGCAACTGCTGAATGAGAA	GGTGACTATCAGCAACCATCC
GAPDH	NM_204305	GTGCTGGCATTGCACTGAA	CACAACACGGTTGCTGTATCC
GH	NM_204359	TCCCAGGCTGCGTTTTGTTA	TGAAAACCGAAGCAGCTCCA
GPR39	NM_001080105	AGAGCTCCAGGAGACAGATCA	TCTGGTTTGGCATCCAGCATA
GPT2	XM_015292423	CCAAGGCATACTGCAATCCA	TGCACTTCCTGCTTTGTACC
GZMA	NM_204457	AAAGTCATTCCCCTGCCTACC	AGATTTGTCCCCATCCTGCTAC
HBEGF	NM_204849	AGAGAGCTGGGTGCTCCA	GGATGCTCTACAGGCAGCAA
HPRT1	NM_204848	AACTCCTCGAAGTGTGGGATA	GAGGGCGTATCCAACAACAA
IFI6	NM_001001296	CGTTCCTTCTGGAGGGACTAC	GGAGATCCCACTGCTGGTAAA
IFIT5	NM_001320422	TCCAGGTGAAATTGACAGGAGAA	GCCCATGTGGTAGTAGATCCAA
IFNLR1	XM_004947908	TTGGGAAGCCGGATCTGAA	TGCAGTGGTGTCGATAGTCA
IL1B	NM_204524	TGCTTCGTGCTGGAGTCAC	GGCATCTGCCCAGTTCCA
KLF4	XM_001233583	ATCTCAAGGCACACCTGAGAA	GATCGGGCAAACTTCCATCC
LIPA	XM_426515	ACCCTTAAGCCCTCTCAGAA	GAAGTACAGCTCTGCTGGAA
MRPL28	NM_001031359	GGCCTATGGCTTCGACTTCTA	CGTTCGCTTCAAGTCCATCC
MSC	XM_015282880	CAAGAGGACCGCTACGAGAA	GTCGTCCCGAGACCACAA
Mx	NM_204609	CTGTTGCGATGCTGAACAAA	TTAGCAAAACGCTTGTTAGCC
NR4A3	XM_015282405	CCGCTTTCAGAAGTGTCTCA	CCGACCTCTTCTCCCTTTCA
OASL	NM_205041	AGCTTCACAGAACTGCAGAA	TCCTTGTACCAGTGCTTGAC
PDK4	NM_001199909	TCTCCGCTCTCCATCAAGCA	TCTTGTCGCAGGAACGCAAA
PROKR2	NM_001145229	TCTACTAACGCTCTCCTGGCTA	CGTGGTTTCAGTGGGTGAAC
RDM1	NM_204546	GCTTTGTCCAGTGCCTTTCA	TCCTCTGCAGCATGGTTGTA
SAMD9L	XM_004939297	GCTTCCTGCTTGAGCAAAA	TTCTCAGTGTTTGCATTTTCCA
SIK1	NM_204682	GAGCAGCAGAGAGGGAAGAAA	GGAGTAGCTGGTGATGCTGAA
TPPP3	XM_015279191	GACGTGGACATTGTGTTCTCC	TCCAGGGCCTTCTTAAACTCC
TRIM25	NM_001318458	TGCAGTGAATGTGTCTGTGGATA	TGTTTGGGGAAGGCACTGAA
USP18	XM_004937958	CAGTGCCAAGCGTATGACAA	TCCGTAGCTAGTTGTTCCTGAA
XDH	NM_205127	CAGGCTGCTGCATGAATGAA	ATGGCTGGAACTCGGAAGAA
YF5	NM_001030675	GCCGGAACGCTACAACAAAA	CAGGATGTCACAGCCGATCA
ZNFX1	XM_015296679	TGCTGAAGTCTGCTGCTGTA	AACATTGGCCGTTCACTGAC
